# Effect of Lithium Salts on the Properties of Cassava Starch Solid Biopolymer Electrolytes

**DOI:** 10.3390/polym15204150

**Published:** 2023-10-19

**Authors:** Alvaro A. Arrieta, Oriana Palma Calabokis, Jorge Mario Mendoza

**Affiliations:** 1 Department of Biology and Chemistry, Universidad de Sucre, Sincelejo 700001, Colombia; 2 Faculty of Engineering and Basic Sciences, Fundación Universitaria Los Libertadores, Bogota 111221, Colombia; opalmac@libertadores.edu.co; 3 Department of Mechanical Engineering, University of Córdoba, Monteria 230002, Colombia; jorge.mendoza@correo.unicordoba.edu.co

**Keywords:** cassava starch, solid biopolymer electrolytes, electrochemistry, crystallinity, lithium salts

## Abstract

This study evaluates the effect of lithium salts on the structural, electrochemical, and thermal properties of cassava starch solid biopolymer electrolytes (SBPEs). Films of SBPEs were synthesized using plasticizing agents and lithium salts (LiCl, Li_2_SO_4_, and CF_3_LiSO_3_) via thermochemical method. The SBPEs with lithium salts exhibited characteristic FTIR bands starch, with slight variations in the vibration oxygen-related functional groups compared to salt-free biopolymer spectra. The R_COH/COC_ index (short-range crystallinity) was higher in the films synthesized without lithium salt and the lowest value was established in the films synthesized with Li_2_SO_4_. Thermal degradation involved dehydration between 40 to 110 °C and molecular decomposition between 245 to 335 °C. Degradation temperatures were close when synthesized with salts but differed in films without lithium salt. DSC revealed two endothermic processes: one around 65 °C linked to crystalline structure changes and the second at approximately 271 °C associated with glucose ring decomposition. The electrochemical behavior of the SBPEs varied with the salts used, resulting in differences in the potential and current of peaks from the redox processes and its conductivity, presenting the lowest value (8.42 × 10^−5^ S cm^−1^) in the SBPE films without salt and highest value (9.54 × 10^−3^ S cm^−1^) in the films with Li_2_SO_4_. It was concluded that the type of lithium salt used in SBPEs synthesis affected their properties. SBPEs with lithium triflate showed higher molecular ordering, thermal stability, and lower redox potentials in electrochemical processes.

## 1. Introduction

Solid polymer electrolytes (SPEs) are materials used in the battery and supercapacitor industry. These materials consist of polymers that have the ability to conduct ions in a similar way to conventional liquid electrolytes, but without the risks associated with spillage or corrosion. SPEs have several advantages, such as higher chemical and thermal stability, charge capacity, resulting in higher energy density and longer device life. Consequently, SPEs are gaining increasing attention in the research and development of energy storage technologies. [1].

Poly(propylene oxide), poly(vinyl alcohol), poly(vinyl acetate), and polyacrylic acid are some of the polymers used for the synthesis of SPEs and have shown excellent properties to be employed in electrochemical devices, either in pure form or combined with other substances [2,3,4,5]. However, SPEs generate some environmental drawbacks due to their chemical composition and their production processes. Many of these polymers contain toxic components that can be harmful to the environment and human health. In addition, the production process of these materials often involves the use of organic solvents, which can be polluting and generate toxic emissions [6]. Therefore, in recent years, work has been carried out on the search for environmentally friendly SPEs that have suitable properties for their application in technological devices.

Solid biopolymer electrolytes (SBPEs) have become attractive alternatives, since they are based on biodegradable and renewable materials, such as starches [7], celluloses [8], and chitosan [9], among others. Cellulose and starch are the most abundant polysaccharides in nature and can be extracted from a wide variety of low-cost natural sources. Starch, in particular, has proven to be a material with excellent physicochemical properties and attractive functional qualities in the formulation of SBPEs. The low viscosity of starch, attributed to its semicrystalline structure, enables greater ion mobility, resulting in higher ionic conductivity [10]. Additionally, starch-based SBPEs offer favorable mechanical and electrochemical properties similar to conventional SPEs, which makes them an interesting option for applications in different sectors. One of the biggest challenges in the research of SBPEs is to improve their ionic conductivity and stability. This has been addressed by incorporating additives into biopolymers or modifying their chemical structure. In addition, new types of biopolymers are being investigated to increase their conductivity and stability. In this sense, the use of Li^+^ conducting pectin in the development of SBPE with a conductivity of 3.44 × 10^−3^ S cm^−1^ [11] and of SBPE elaborated from iota-carrageenan with LiCl via solution casting approach which reached conductivities of 5.33 × 10^−3^ S cm^−1^ has recently been reported [12].

In general, SBPEs have the potential to improve the sustainability and safety of electronic devices, making them an area of scientific and technological interest for the industry. SBPEs can be easily prepared as solids or semi-solids. These SBPEs possess desirable properties, including electrochemical stability, ease of processability, flexibility, and high energy density. As a result, they hold great promise for use in various electrochemical devices, such as supercapacitors, batteries, solar cells, and electroluminescent devices, among others [13].

Starch is a natural biopolymer that can be extracted from different sources, such as potatoes, corn, cassava, or wheat. Starch is a complex carbohydrate that is made up of two types of macromolecules, amylose formed by α-D-glucopyranosyl (glucose) units with a mainly linear structure and amylopectin formed by branched chains. The proportion of amylose and amylopectin in starches varies depending on the botanical origin and range between 20 and 25% amylose and 80 and 75% amylopectin [14]. Although starch biopolymer has many applications, it also presents some challenges in its use. For example, the high crystallinity of starch limits its processing and mechanical resistance, so additives, plasticizers, and chemical modifications are used to improve its properties [15,16]. Additionally, humidity and temperature can affect the stability of starch biopolymer, which can limit its use. In general, starch biopolymer is a promising and sustainable alternative in multiple industries, but improvements to its properties are necessary to enhance and expand its use.

Starch has been used in the preparation of SBPEs in their pure form or mixed with some compounds [17]. The synthesis of starch SBPEs of different botanical origins was reported and the effect of this factor on its properties was studied [18], the synthesis of starch SBPEs using potato starch doped with glutaraldehyde and polyethylene glycol as plasticizers and NaI as lithium salt [19], tamarind seed starch was used with CF_3_LiSO_3_ salt [20], and rice starch with different iodine salts (NH_4_I, LiI, and NaI) [21], among others. Additionally, the combination of starch with other compounds has been reported to improve the properties of the resulting SBPEs, for example, the combination of starch with chitosan [22], cellulose [23], polyaniline [24], polypyrrole [25], poly(vinyl alcohol) [26], etc.

Lithium salts are the most used to generate the charge carriers in the polymer matrix of SBPEs, because the conductivity depends mainly on ionic mobility and lithium is the lightest metal, smaller in size, and with the lowest redox potential. Additionally, it is important to mention that lithium ions are not very chemically active and therefore have a low environmental impact and are widely distributed in different ecosystems naturally. However, lithium in its pure form is a highly reactive element and can react with different compounds, making it an environmentally unfriendly material [27,28]. Therefore, SBPEs that contain lithium in the ionized form do not represent a risk to ecosystems due to their biodegradability and low environmental impact. This work seeks to determine the effect of the type of lithium salts (Li_2_SO_4_, LiCl, and CF_3_LiSO_3_) used in the synthesis of cassava starch SBPEs on the thermal, structural, and electrochemical properties, determined by thermogravimetric analysis, differential scanning calorimetry, infrared spectroscopy, cyclic voltammetry, and electrochemical impedance spectroscopy.

## 2. Materials and Methods

### 2.1. Reagents and Materials

The reagents used in the different experiments carried out in this work were: glycerol (C_3_H_8_O_3_; 99.5%; Merck, Darmstadt, Germany), polyethylene glycol 400 (C_2_nH_4_n_+2_On_+1_; 98.5%; Merck), glutaraldehyde (C_5_H_8_O_2_; 70.0%; Aldrich, St. Louis, Missouri, USA), lithium chloride (LiCl; 99.5%; Aldrich), lithium trifluoromethanesulfonate/i.e., Lithium triflate (CF_3_LiSO_3_; 99.9%; Aldrich), and lithium sulfate (Li_2_SO_4_; 99.5%; Aldrich). The water used was of ultrapure quality obtained by filtration in a Stakpure—OmniaTap 6 water purification system. The starch used was extracted in the laboratory from tubers of the *Manihot esculenta* Crantz variety. A total of 500 g of tubers was washed and peeled. The peeled tubers were disintegrated by blending in an industrial processor. The disintegrated material was dispersed in 1 L of water and stirred for 15 min and then filtered through a muslin cloth. The liquid was left to rest for 12 h and decanted to obtain the starch precipitate, which was washed three times with distilled water. The starch was dried in an oven for 24 h at 50 °C. The obtained starch was crushed and sieved through a 200 µm mesh sieve; the starch powder was bright white. The extracted starch was characterized via infrared spectroscopy (FTIR) and the purity was determined at 99.3%, using the official methods of the International Association of Analytical Chemists.

### 2.2. Synthesis of Cassava Starch SBPE Films

The synthesis of cassava starch SBPE films was carried out via the thermochemical method [6,13]. A total of 3 g of starch was dispersed in 100 mL of water by constant stirring at 1500 rpm. The dispersion was heated to 70 ± 5 °C for 15 min with constant stirring to gelatinize it, then it was allowed to cool to room temperature. The dispersion was again stirred at 1500 rpm. and the plasticizers and salts were added; glycerol (1.5 g), polyethylene glycol (1.5 g), glutaraldehyde (1.0 g), and the dopant salt (1.5 g). The salts studied and the codes used to label the cassava starch SBPEs were lithium triflate (code: SCB-CF_3_LiSO_3_), lithium sulfate (code: SCB-Li_2_SO_4_), lithium chloride (code, SCB-LiCl), and salt-free films (code: SCB-SF) were also prepared.

The mixtures were heated at 70 ± 5 °C for 15 min with constant stirring. Then, they were allowed to cool to room temperature and were poured into Teflon Petri dishes to heat them in an oven at 70 °C for 48 h. After the heating process, they were allowed to rest for 24 h before the films were detached from the Petri dishes.

### 2.3. Characterization of Cassava Starch SBPE Films

#### 2.3.1. Characterization via Fourier Transform Infrared Spectrometry

Infrared spectra were recorded using Fourier Transform Infrared Spectrometry (FTIR) with a Perkin-Elmer Spectrum Two instrument equipped with an ATR (attenuated total reflectance) with diamond/ZnSe crystal. The spectra were recorded at room temperature, with wavenumber range of 400 to 4000 cm^−1^, mirror speed of 0.4 cm/s, and resolution of 4 cm^−1^.

#### 2.3.2. Characterization via Thermal Analysis

The thermal analysis was carried out with a Perkin-Elmer STA 6000 instument, with flow of 20 mL/min of high purity dry N_2_ (99.9%), temperature range of 40 to 600 °C, and sweep speed of 10 °C/min. The samples were weighed in a ceramic pan and the weights were in the range of 1.5 to 2 mg. The thermogravimetric analysis (TGA) of the samples was recorded with the derivative of the percentage of weight loss of the samples and the measurements were taken simultaneously via difference scanning calorimetry (DSC) and were recorded using the flow of heat of the samples.

#### 2.3.3. Characterization by Cyclic Voltammetry

Cyclic voltammetry was carried out with a Gamry 1010E potentiostat/galvanostat, using a cell for solid measurements made up of two 1 × 1 cm steel sheets, between which the samples were located. This type of cell was used to evaluate the redox behavior of the samples. The voltammetric signals were recorded in potential range of −2 to 2 V, sweep speed of 10 mV/s, and the potentials were measured with respect to the open circuit potential (0.10 V).

#### 2.3.4. Characterization via Electrochemical Impedance Spectroscopy

Electrochemical impedance spectroscopy was performed using a cell for solid samples made up of two 1 × 1 cm stainless steel sheets, between which the SBPEs samples were sandwiched. Measurements were carried out on 1 × 1 cm samples with a Gamry 1010E potentiostat/galvanostat. To record the impedance spectra, a frequency range of 2.0 MHz to 10 mHz was used with r.m.s AC of 10 mV and open circuit potential (OCP) as reference potential (0.15 V).

## 3. Results and Discussion

### 3.1. FTIR Characterization of Cassava Starch SBPE Films

**Figure 1:**
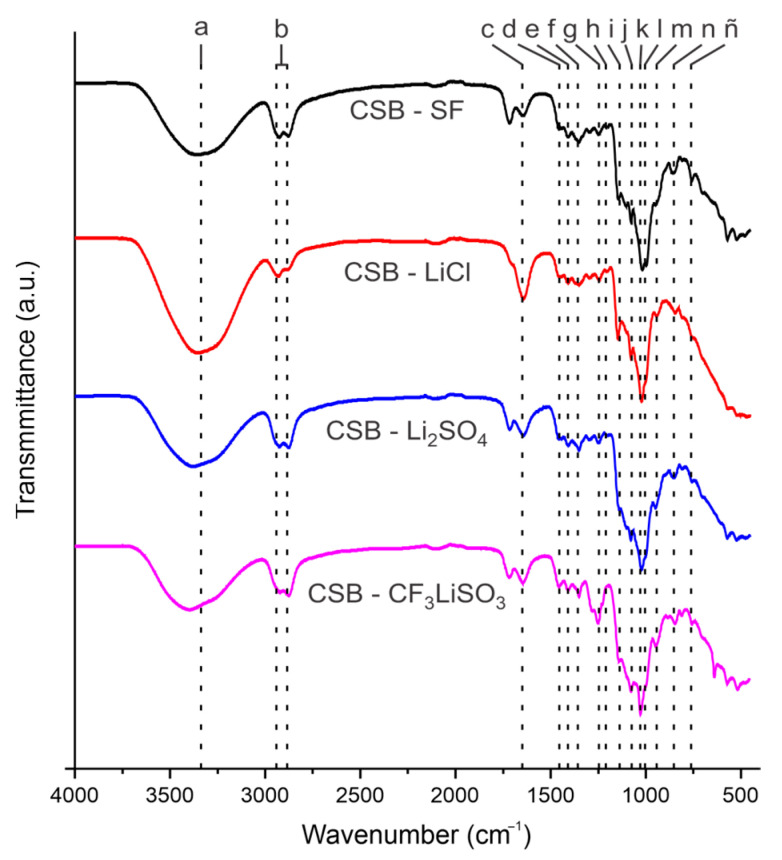
FTIR spectra of cassava starch SBPE films: CSB-SF, CSB-LiCl, CSB-Li_2_SO_4_, and CSB-CF_3_LiSO_3_. The dotted lines indicate the vibration assignments to the different bands denoted with lowercase letters that are summarized in Table 1.

All the cassava starch SBPE films were easily detached from the Petri dishes and were stable against manual traction, presenting a light amber color with medium transparency. Figure 1 shows the FTIR spectra of cassava starch SBPE films CSB-SF, CSB-CF_3_LiSO_3_, CSB-Li_2_SO_4_, and CSB-LiCl.

In SBPEs, electrical conductivity is generated by the movement of ions (charge carriers). Consequently, the interactions between the ions of the dissociated salt and the biopolymeric molecules are of great importance. The possible interactions and effects of the type of salt used in the preparation of SBPEs can be evidenced using infrared spectroscopy [29,30,31]. Figure 1a shows the FTIR spectrum obtained from SBPE-FS films, where the characteristic bands of starch reported in the literature are observed [32,33]. The CSB-SF spectrum presented a broad band at 3364 cm^−1^ due to OH stretching, two peaks at 2926 and 2878 cm^−1^ due to CH stretching, and at 1649 cm^−1^ due to the O-H bending of water. In the bands at 1456, 1407, 1350, 1247, and 1201 cm^−1^, the vibrations assigned to CH bending, OH bending, COH bending, CH_2_OH-related modes, and COH deformation, respectively, were evident. The CO antisymmetric bridge stretching group was observed at 1146 cm^−1^. Additionally, in the region from 1120 to 700 cm^−1^, the bands of COH stretching antisymmetrically in plane ring stretching, the bending of C-OH, the COC ring vibration of carbohydrate, COH solvated, C-H, and CH_2_ were observed at positions 1103, 1077, 1018, 995, 844, and 757 cm^−1^, respectively. In the FTIR spectra of the cassava starch SBPE films with lithium salts, the characteristic bands of starch predominated. The characteristic vibration bands of the functional groups in salts, such as: S=O stretching at 1040 cm^−1^, SO_3_ stretching at 1250 cm^−1^ and CF_3_ stretching at 1225 cm^−1^ [34,35], were not clearly discernible due to their overlap with the vibrational bands of starch. However, they presented some differences in width, position, and intensity in relation to the bands observed in CSB-SF.

The vibration assignments corresponding to the bands observed in the cassava starch SBPEs are summarized in Table 1. It could be observed that the vibrations related to the oxygen atoms presented variations. Oxygen atoms can act as electron donors, so the vibrations related to these atoms revert greater interest in this study. The free electrons of the oxygen atoms can form dative bonds with the lithium ions from the added salts, so biopolymer–Li complexes could be formed [30,31].

In order to study the effect of the biopolymer–ion molecular interactions on the FTIR spectroscopic response of SBPEs, four vibrational regions of the spectrum were evaluated. The region of the band assigned to the O-H stretching showed variation between the CSB-SF (3364 cm^−1^) and those registered in the SBPEs synthesized with salts, showing higher values with the salts CF_3_LiSO_3_ (3398 cm^−1^) and Li_2_SO_4_ (3373 cm^−1^) and lower with LiCl (3350 cm^−1^).

The region of the vibrations of the band corresponding to the O-H bending of the water in the biopolymer matrix occurred at 1649 cm^−1^ in CSB-SF and these bands showed slightly different positions in the SBPEs with salts, showing values of 1647 cm^−1^ with the CF_3_LiSO_3_ salt, 1642 cm^−1^ with the Li_2_SO_4_ salt, and 1641 cm^−1^ with the LiCl salt. Variations in the region between 1270 to 1140 cm^−1^ were also evident in the bands assigned to CH_2_OH, COH, and COC, which were located in the CSB-SF spectrum at 1247, 1201, and 1146 cm^−1^, respectively, while in the spectra of SBPEs with salts were located at 1250, 1223, and 1141 cm^−1^ for the CSB-CF_3_LiSO_3_, in the CSB-Li_2_SO_4_ spectra, were located at 1247, 1203, and 1142 cm^−1^ and for CSB-LiCl were shown at 1245, 1204, and 1145 cm^−1^. The region of the bands assigned to COH and COC present in CSB-SF at 1018 and 995 cm^−1^, showed different positions in the CSBs with salts; in CSB-CF_3_LiSO_3_, they were shown at 1028 and 997 cm^−1^, in CSB-Li_2_SO_4_ at 1021 and 998 cm^−1^, and in CSB-LiCl at 1021 and 997 cm^−1^. As previously mentioned, the variations in the positions of the different bands associated with oxygen atoms in the biopolymeric matrix can show the interactions with the different salts used, not only with the Li ion, but possibly with the anionic part of the salt in the matrix [36,37].

The FTIR spectra of SBPEs allowed establishing differences in the crystallinity of the molecular structure. As has been reported, the bands at 1020 and 995 cm^−1^ can be related to the crystallinity and water content of starch-based biopolymers [29,36,37]. The relative intensity of the bands of COC and COH vibrations can be related to the molecular arrangement of starch biopolymers, so it can be considered a short-range crystallinity index [29].

**Figure 2:**
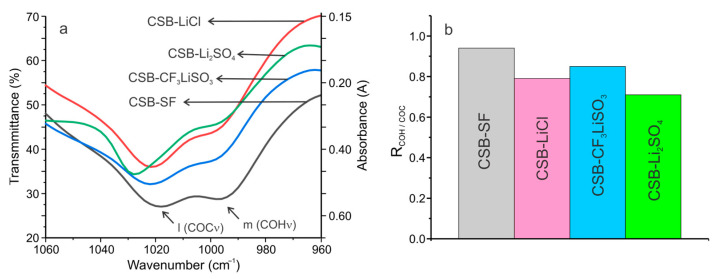
(**a**) FTIR region from 1060 to 960 cm^−1^ of the cassava starch SBPEs spectrum; (**b**) R_COH/COC_ index of short range crystallinity.

Figure 2a shows the infrared region of 1060 and 960 cm^−1^. Differences can be seen in the intensities and positions of the bands assigned to COH solved and COC ring vibrations in the cassava starch SBPEs. In addition, their relative intensities were also variable. Figure 2b shows the graph of the R_COH/COC_ index of short range crystallinity, calculated for each of the cassava starch SBPEs. It can be observed that the R_COH/COC_ index decreased in the order CSB-SF, CSB-CF_3_LiSO_3_, CSB-LiCl, and CSB-Li_2_SO_4_, so the results obtained indicate that the ordering in the biopolymeric molecules (i.e., crystallinity) of the SBPE films decreased in the same order.

The lithium salts used in the synthesis of SBPEs are dissociated in the biopolymer matrix into its cationic and anionic part $\left(LiCl={Li}^{+}/{Cl}^{-};{Li}_{2}{SO}_{4}={2Li}^{+}/{SO}_{4}^{2-};{CF}_{3}{LiSO}_{3}={Li}^{+}/{{CF}_{3}SO}_{3}^{-}\right)$. Li^+^ cations can interact with the oxygen atoms of glucose units in the biopolymer chain of starch to form biopolymer–Li complexes through the coordination of biopolymer oxygen atoms and the Li^+^ cation, which affect molecular packaging and therefore affect its crystallinity. Additionally, the anionic component of the salts, without a doubt, can also affect the biopolymeric structure and its properties because it can generate molecular interactions or/and hydrogen bonds with the protons of the hydroxyl groups in the glucose units or the plasticizers [30,36]. These interactions can occur because the oxygen atoms in the sulfate anions (${SO}_{4}^{2-}\;\mathrm{and}\;{CF}_{3}{SO}_{3}^{-}$) can easily interact with the hydrogen atoms of the OH groups present in the biopolymeric matrix (starch and plasticizers). In addition, it has been reported that ${Cl}^{-}$ ions can electrostatically interact with OH groups in biopolymeric matrices [30].

### 3.2. Thermal Characterization of Cassava Starch SBPE Films

**Figure 3:**
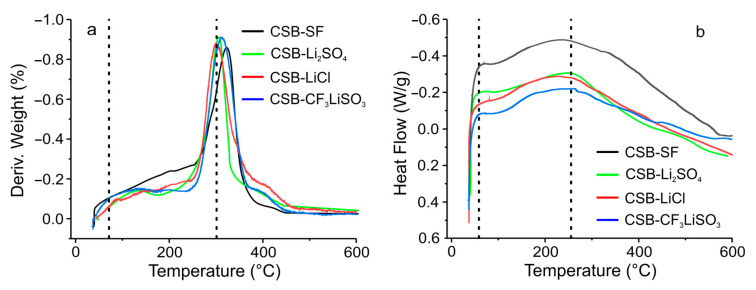
Thermal analysis of cassava starch SBPE films carried out via (**a**) thermogravimetric analysis (Dotted lines indicate weight loss processes) and (**b**) differential scanning calorimetry (Dotted lines indicate endothermic processes).

Figure 3 presents the curves of thermal analysis performed by thermogravimetric analysis (TGA) and differential scanning calorimetry (DSC). The thermal decomposition of the cassava starch SBPE films presented in Figure 3a showed a process of weight loss due to the dehydration of the films in the region of 40 to 110 °C in all the films. The second weight loss process was observed between 245 and 335 °C with peaks at 298.3, 298.9, 299.5, and 308.1 °C for CSB-LiCl, CSB-Li_2_SO_4_, CSB-CF_3_LiSO_3_, and CSB-SF, respectively. This weight loss was due to the thermal decomposition of the films and started after the dehydration process had been completed, so the loss of water did not affect the decomposition process of the SBPE films. The temperature recorded in the decomposition process of CSB-SF was slightly higher (approximately in 9.8 °C) than that of the films synthesized with the different lithium salts, which did not present differences between them, indicating that the type of salt did not have a significant effect on the thermogravimetric behavior of the SBPEs studied.

Figure 3b shows the differential scanning calorimetry curves of cassava starch SBPE films. It was possible to observe in all the recorded curves, that two endothermic processes occurred. One due to short-range crystallinity breakdown produced during retrogradation, presented at about 65 °C. The temperatures of this band in the different cassava starch SBPEs were 63.1, 65.6, 67.2, and 70.2 °C for CSB-Li_2_SO_4_, CSB-LiCl, CSB-CF_3_LiSO_3_, and CSB-SF, respectively. When calculating the total endothermic heat by integrating the curve, values of 48.4, 54.3, 59.9, and 68.1 J/g were obtained for CSB-Li_2_SO_4_, CSB-LiCl, CSB-CF_3_LiSO_3_, and CSB-SF. As has been reported, this enthalpy is related to the extension of the ordered structures in the SBPEs, so this result indicates that the CSB-SF films presented more ordered structures, followed in decreasing order by CSB-CF_3_LiSO_3_, CSB-LiCl, and CSB-Li_2_SO_4_. This result is consistent with that obtained by the studies carried out via FTIR spectrometry. The second process was observed in the band located at approximately 271 °C in all the films. This endothermic process corresponds to the breakdown of the glucose ring of starch chains [38,39,40]. This result showed that this endothermic process was not significantly affected by the use of the lithium salt or the type of salt used in the synthesis of cassava starch SBPEs.

### 3.3. Electrochemistry Characterization of Cassava Starch SBPE Films via Cyclic Voltammetry

**Figure 4:**
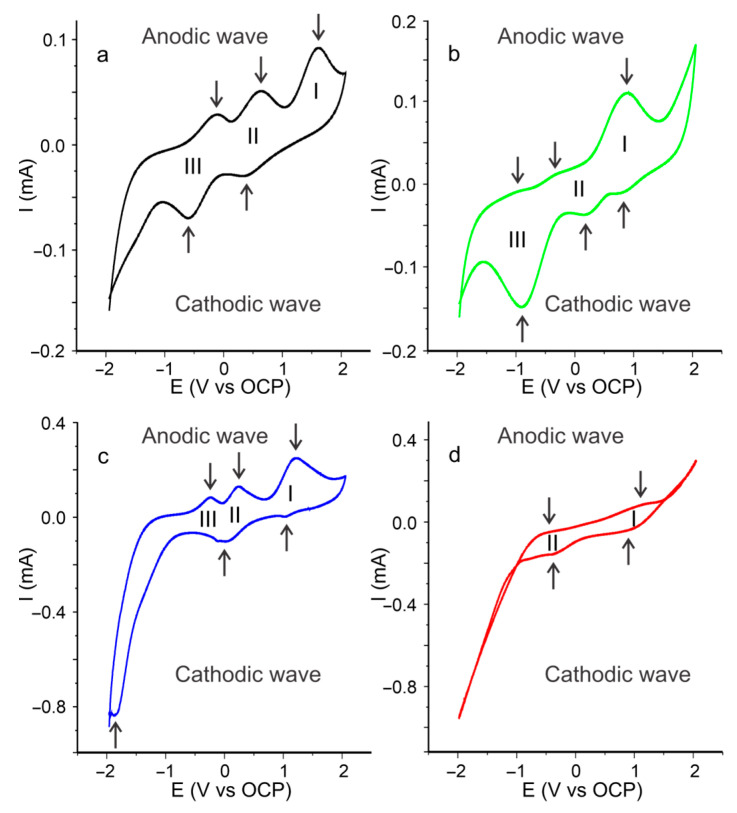
Voltammetric signals of cassava starch SBPE films (**a**) CSB-SF, (**b**) CSB-CF_3_LiSO_3_, (**c**) CSB-Li_2_SO_4_, and (**d**) CSB-LiCl. Roman numbers indicate the redox processes.

The electrochemical study carried out using cyclic voltammetry on the cassava starch SBPE films, allowed to evaluate the redox processes that take place in a potential range of −2.0 to +2.0 V. Figure 4 presents the voltammograms of the cassava starch SBPE films. It was possible to see that the voltammograms presented in all cases, redox processes with anodic (oxidation) and cathodic (reduction) peaks. The SBPEs synthesized without salt and with the Li_2_SO_4_ and CF_3_LiSO_3_ salts presented signals with three well-defined processes, while the signal of the SBPE synthesized with LiCl showed a signal with two less defined redox processes. Oxidation and reduction processes in starch SBPE films take place in glucose units. Several authors have reported that glucose can undergo three oxidation and reduction processes [41,42]. The proposed mechanism involves a first phase of the dehydrogenation of glucose in its C1 (process III); followed by the oxidation of glucose by hydroxyl ions (OHs), which are products of the dissociation of water molecules (process II); and a subsequent oxidation reaction of glucose by the metal oxides present on the surface of the electrode. These reactions are quasi-reversible, so oxidation peaks can be observed in the anodic scan (positive currents) and reduction peaks in the cathodic scan (negative currents).

The voltammetric signal of CSB-SF (Figure 4a) presented three redox processes; a process with a single peak in anodic sweep at 1.52 V, a second process composed of a redox couple at 0.57 V in the anodic wave and 0.31 V in the cathodic wave, and a third process with anodic and cathodic peaks at −0.19 and −0.66 V, respectively. The voltammetric signal obtained from the CSB-CF_3_LiSO_3_ films (Figure 4b) presented in its anode wave three processes at 0.86, −0.22, and −0.87 V, while in the cathode wave, the peaks were located at 0.80, 0.13, and −0.91 V. For its part, the CSB-Li_2_SO_4_ film (Figure 4c) exhibited in its signal three redox processes with anodic and cathodic redox pairs, the first process presented the anodic peak at 1.16 V and the cathodic peak at 1.00 V, the second process showed the anodic peak at 0.19 V and the cathode at −0.04 V, and the third process the anodic and cathodic peaks were located at −0.22 V and −1.88 V, respectively. The CSB-LiCl voltammetric signal (Figure 4c) presented two anodic peaks at 1.05 and −0.66 V and two cathodic peaks at 0.81 and −0.45 V.

The voltammetric signals of cassava starch SBPE films presented differences not only in the shape and positions (i.e., peak potentials) but also in the intensities (i.e., peak currents), which is related to their conductivity. Table 2 shows the values of the potentials and peak currents (Ep and Ip, respectively) of the oxidation and reduction of the processes in the voltammetric signals of cassava starch SBPE films. The CSB-Li_2_SO_4_ voltammetric signal presented anode peaks with higher current intensity (0.248 µA), followed in descending order by CSBLiCl (0.155 µA), CSB-CF3LiSO3 (0.113 µA), and CSB-SF (0.096 µA).

The differences in the conductivities of the SBPEs can be related to the degree of molecular ordering, since in more ordered or crystalline structures, there can be less movement of charges due to their reduced fluidity, while in amorphous structures, fluidity enables greater charge mobility. Therefore, the intensities in the signals (i.e., currents) showed a consistent behavior with what was established in the results obtained from the R_COH/COC_ index of short range crystallinity and the thermal decomposition processes established in the TGA and DSC analysis. These differences generated by the salts in the SBPEs can affect the electrochemical behavior shown in the waves or voltammetric signals, because the morphology and molecular packing can influence the electrochemical kinetics, generating variations in the position and width of the peaks.

### 3.4. Electrochemistry Characterization of Cassava Starch SBPE Films by Impedance Spectroscopy

**Figure 5:**
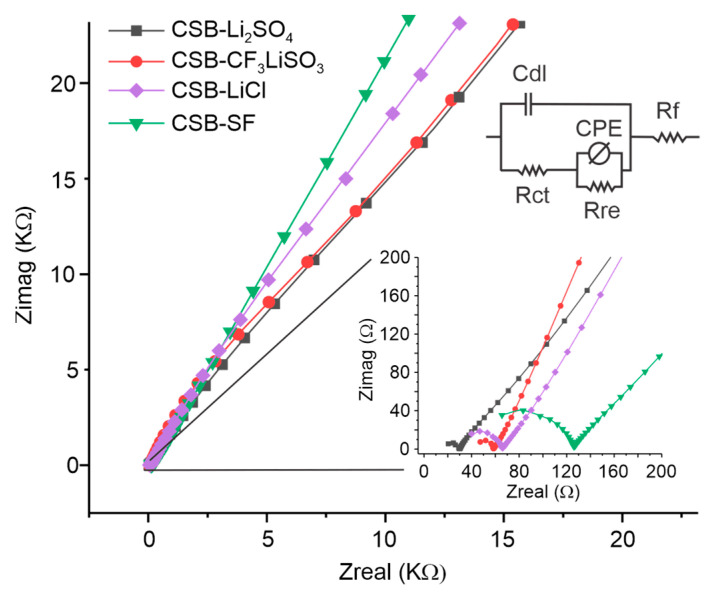
Nyquist plot of cassava starch SBPE films with equivalent circuit.

Figure 5 presents the Nyquist plot recorded through electrochemical impedance measurements carried out on cassava starch SBPE films and the equivalent circuit corresponding to the obtained impedance behavior.

The Nyquist plot consisted of a semicircle at high frequencies (zoomed graph), which can be attributed to the parallel phenomena of capacitance and resistance at the electrode/SBPE interface, which are represented in the equivalent circuit as *Cdl* (double-layer capacitance) and *Rct* (charge transfer resistance), respectively. Continuous to the semicircle, an inclined line of positive slope can be observed, which begins gently curved at medium frequencies and ends straight at low frequencies. This behavior is related to the abnormal transport of electrons towards the biopolymer matrix and may be due to the occurrence of electrochemical oxidation and reduction processes. In the equivalent circuit, these processes are described by a parallel system composed of a resistance (*Rre*) and a constant phase element (*CPE*). Additionally, the intersection of the semicircle with the real axis at low frequency allows the determination of the values of the bulk resistance of cassava starch SBPE films (*Rf*).

The bulk ionic conductivity (σ) of SBPE films can be determined by the equation σ=l/Rf A, where *l* is the thickness of the film, *Rf* is the bulk resistance, and *A* is the contact area between the films and the electrodes. The bulk ionic conductivities of cassava starch SBPE films were 9.54 × 10^−3^, 1.89 × 10^−3^, 4.59 × 10^−4^, and 8.42 × 10^−5^ S cm^−1^, corresponding to CBS-Li_2_SO_4_, CSB-CF_3_LiSO_3_, CSB-LiCl, and CSB-SF, respectively. These trends in the conductivity values correspond to the trend observed in the current intensities (Ip) of the voltammetric signals. Table 3 presents the ionic conductivities of the studied SBPEs and includes some data on the ionic conductivities of starch SBPEs reported by other authors for comparison.

## 4. Conclusions

The effect of the lithium salt used in the thermochemical synthesis of cassava starch SBPEs with the salts CF_3_LiSO_3_, Li_2_SO_4_, and LiCl on the structural, thermal, and voltammetric properties was evaluated. Infrared spectroscopy allowed to establish differences in the bands related to the oxygen atoms of cassava starch SBPEs due to their interaction with the ions released by the dissociation of the salts. Additionally, the spectra allowed establishing relationships with the degree of crystallinity through the R_COH/COC_ index of short range crystallinity, showing greater crystallinity in the films synthesized without salt (CSB-SF), followed in descending order by those synthesized with CF_3_LiSO_3_, LiCl, and Li_2_SO_4_. The thermal analysis carried out allowed us to conclude that the addition of salt and the type of salt does not significantly affect the dehydration temperatures observed in the range of 40 to 110 °C and decomposition temperatures between the 245 and 335 °C of cassava starch SBPEs. On the other hand, the temperatures of endothermic processes evidenced by DSC analysis presented very close values in all the films of cassava starch SBPEs. However, the enthalpy values of the endothermic process related to crystallinity showed the same order obtained by the R_COH/COC_ index of short range crystallinity. Cyclic voltammetry performed on the cassava starch SBPE films showed that the salt used had an important effect on the redox processes recorded, presenting different Ep and Ip values with each of the salts studied. It was possible to observe a trend of the intensity of the signals (i.e., peak currents) in relation to the use of salts, which corresponded to the degrees of crystallinity observed in the thermal analysis and via FTIR. The conductivity of the films, determined via impedance spectroscopy, showed the highest conductivity value in the SBPE films synthesized with Li_2_SO_4_ and the lowest value in the films synthesized without salt.

## Figures and Tables

**Table 1:** FTIR vibration bands of cassava starch SBPEs.

Band	Assignments	Wavenumber (cm^−1^)			
		CSB-SF	CSB-CF_3_LiSO_3_	CSB-Li_2_SO_4_	CSB-LiCl
a	O-H stretching	3364	3398	3373	3350
b	C-H stretching	2926, 2878	2922, 2876	2925, 2877	2931, 2877
c	O-H (water) bending	1649	1647	1642	1641
d	C-H bending	1456	1456	1456	1456
e	O-H bending	1407	1407	1407	1407
f	COH bending/S=O stretching	1350	1350	1350	1350
g	CH_2_OH related modes/SO_3_ stretching	1247	1250	1247	1245
h	COH deformation	1201	1223	1203	1204
i	CO antisymmetric bridge stretching	1146	1141	1142	1145
j	COH antisymmetric stretching in plane ring	1103	1102	1103	1104
k	C-OH bending	1077	1077	1078	1077
l	COC ring vibration of carbohydrate/ CF_3_ stretching	1018	1028	1021	1021
m	COH solved	995	997	998	997
n	C-H bending modes	844	844	843	845
ñ	CH_2_ rocking	757	757	757	757

**Table 2:** Values of potentials and peak currents of the anodic and cathodic electrochemical processes of cassava starch SBPE films.

Process	Peak		Solid Biopolymer Electrolytes			
			CSB	CSB-CF_3_LiSO_3_	CSB-Li_2_SO_4_	CSB-LiCl
I	Anodic	Ep (V)	1.52	0.86	1.16	1.05
		Ip (µA)	0.096	0.113	0.248	0.155
	Cathodic	Ep (V)	-	0.80	1.00	0.81
		Ip (µA)	-	−0.010	−0.188	−0.013
II	Anodic	Ep (V)	0.57	−0.22	0.19	-
		Ip (µA)	0.056	0.016	0.117	-
	Cathodic	Ep (V)	0.31	0.13	−0.04	-
		Ip (µA)	−0.028	−0.038	0.050	-
III	Anodic	Ep (V)	−0.19	−0.87	−0.22	−0.66
		Ip (µA)	0.032	0.006	0.074	0.004
	Cathodic	Ep (V)	−0.66	−0.91	−1.88	−0.45
		Ip (µA)	−0.070	−0.148	−0.848	−0.128

**Table 3:** Ionic conductivity of starch SBPE films with different salts.

SBPEs	Ionic Conductivity (S cm^−1^)
CBS–Li_2_SO_4_	9.54 × 10^−3^
CSB–CF_3_LiSO_3_	1.89 × 10^−3^
CSB–LiCl	4.59 × 10^−4^
CSB–SF	8.42 × 10^−5^
Starch–LiPF_6_ [43]	1.47 ×10^−4^
Starc/Ch–KI [44]	4.65 × 10^−4^
Starch/Ch–NH_4_I–gly [45]	1.28 × 10^−3^

## Data Availability

The data that support this work and its results are not available to be shared because they are under confidentiality agreements. Access to the data can be requested through an official document.
